# Well Met: RIPK1 senses S-adenosyl methionine to ensure cell survival

**DOI:** 10.1093/lifemeta/loaf033

**Published:** 2025-08-28

**Authors:** Piyush Sharma, Douglas R Green

**Affiliations:** Department of Immunology, St. Jude Children’s Research Hospital, Memphis, TN 38105, the United States; Department of Immunology, St. Jude Children’s Research Hospital, Memphis, TN 38105, the United States

**Receptor interacting kinase-1 (RIPK1) plays central roles in multiple cell death and signal transduction pathways. A recent paper describes a role for RIPK1 in sensing the levels of s-adenosyl methionine, a metabolic product of the amino acid methionine (Met), required for protein and nucleic acid methylation. Methylation of RIPK1 restricts its activity, and when the levels of Met are low, the absence of this methylation promotes RIPK1-dependent apoptosis *in vitro* and *in vivo.***

Optimal metabolic status is a hallmark of cellular survival. Aberrations in the cellular metabolome, whether due to insufficient nutrient availability or altered metabolic pathways, can lead to cellular stress and cell death. While cells can synthesize certain amino acids for their use, they rely on extracellular availability for essential amino acids such as methionine (Met). Met is crucial not only for protein synthesis but also as the sole precursor of the S-adenosyl methionine (SAM) pathway, which is responsible for methylation of proteins and nucleotides. Despite the significance of SAM, the mechanisms by which cells sense and regulate SAM levels to sustain cellular functions are not entirely understood. Chen *et al*. found that receptor-interacting protein kinase-1 (RIPK1) functions as a sensor for SAM levels, promoting cell death upon Met deprivation [1].

RIPK1 serves as a pivotal mediator in inflammation and cell death pathways. It contains three domains: the N-terminal kinase domain, the intermediate domain, and the C-terminal death domain (DD). The kinase domain and the intermediate domain are responsible for RIPK1−RIPK3 interactions in the cell death process of necroptosis, while the C-terminal DD interacts with the adapter protein Fas-associated death domain (FADD), leading to caspase-8 activation and apoptosis in some settings.

While it may not be surprising that the absence of a required metabolite can result in cell death, the mechanisms by which this occurs are diverse. Metabolite deprivation can be sensed by the integrated stress response (ISR) kinases [2]. In the case of amino acid deprivation, the kinase general control non-depressible 2 (GCN2) can become active to engage the ISR, and if unresolved, the ISR can promote apoptotic cell death.

Met deprivation has been associated with cell death, initially attributed to the lack of active translation and cellular homeostasis. The Met-derived metabolite, SAM, is required to disrupt the SAM sensor upstream of mTORC1 (SAMTOR)−GAP activity towards Rags complex 1 (GATOR1) interaction, which inhibits the mammalian target of rapamycin complex 1 (mTORC1); thus, SAM allows mTORC1 activation to proceed. Inhibition of mTORC1 can promote apoptotic cell death due to loss of protein expression of the anti-apoptotic protein, myeloid cell leukemia 1 (MCL1) [3]. Therefore, limited availability of Met (and SAM) can promote apoptosis by mTORC1 inhibition and loss of MCL1.

Studies have underscored the use of Met deprivation as a strategy to deprive rapidly proliferating tumor cells of necessary elements for growth, thereby inducing cell death [4]. However, studies have also shown that Met-restricted environments, such as the tumor microenvironment, adversely affect CD8^+^ T cell activation and function [5]. One mechanism whereby limiting Met availability during T cell activation impacts subsequent cell fate is a requirement for arginine methylation of the potassium ion channel, KCa3.1, for optimal calcium signaling, and without such methylation, CD8^+^ T cells rapidly lose function *in vivo* [6].

In their paper, Chen *et al*. found that RIPK1 became activated under conditions of prolonged Met deprivation. The absence of extracellular Met prompted the self-assembly of RIPK1, leading to dimerization of caspase-8 and subsequent apoptosis. Their *in vivo* studies further revealed that an Met-restricted diet induced liver damage, which was ameliorated in *Ripk1*^D138N/D138N^ (kinase-dead RIPK1) mice, thereby indicating a specific role of RIPK1 in Met-deprived conditions.

As mentioned above, RIPK1 can induce two forms of cell death, necroptosis (which is generally an inflammatory and immunogenic form of cell death) or apoptosis (which is not inflammatory and often non-immunogenic). Notably, activation of RIPK1 through Met deprivation results in apoptosis rather than necroptosis, as evidenced by the observation that cells deficient in mixed lineage kinase domain-like protein (MLKL) or RIPK3, both of which are required for necroptosis, exhibited cell death comparable to wild-type cells. Additional experiments ruled out the involvement of two other forms of cell death, ferroptosis and pyroptosis. Furthermore, the authors found that SAM supplementation prevented lethality due to Met deprivation without changes in intracellular Met concentrations, suggesting that a reduction in protein methylation promotes cell death in this setting.

**Figure 1:**
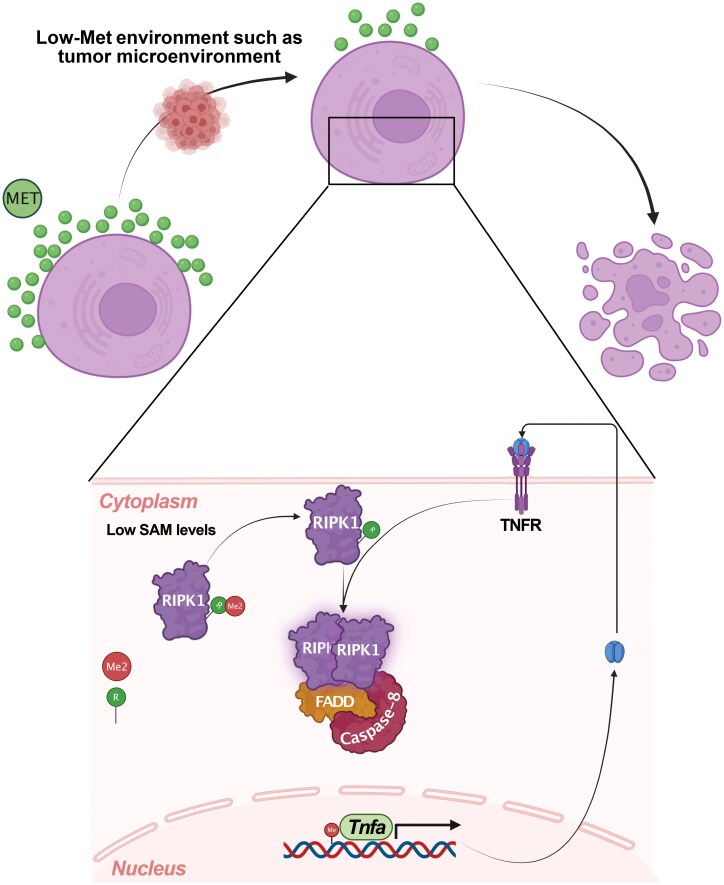
Reduced availability of extracellular Met decreases intracellular SAM levels, resulting in reduced DNA and protein methylation. *Tnfa* promoter hypomethylation leads to an increase in TNF-α autocrine signaling, which, when accompanied by a lack of RIPK1 R606 methylation, causes RIPK1 self-association and activation, initiating the downstream apoptosis signaling cascade via caspase-8. Illustration is created with Biorender.com.

Mechanistically, Chen *et al*. found that Met deprivation creates an inflammatory environment, leading to an increase in tumor necrosis factor (TNF) levels and subsequent RIPK1 activation via autocrine TNF signaling. This activation was abolished in TNF receptor 1-deficient cells. However, they further elucidated that TNF alone did not induce RIPK1 activation; rather, it was contingent upon reduced intracellular SAM levels. Specifically, they found that the Met pathway is responsible for posttranslational methylation of arginine at 606 (R606) in the DD of RIPK1. This finding was further correlated with increased TNF levels, as Met deprivation also resulted in hypomethylation of cytosine−guanine (CpG) residues in the promoter of *Tnfa* (encoding TNF), leading to increased activity prior to RIPK1 activation. Further analysis revealed that RIPK1 R606 is crucial for self-association and kinase activation, as the RIPK1 R606K (arginine to lysine) mutation reduced homo-dimerization of RIPK1. The authors confirmed the physiological role of R606 methylation by generating *Ripk1*^R606K/R606K^ mutant mice, which were resistant to liver damage and inflammation resulting from the Met-restricted diet. These results provide a novel perspective on how metabolic alterations can affect protein−genome and protein−protein interactions, influencing cell and organismal fate (Fig. 1).

Protein arginine methyltransferases (PRMTs) are enzymes responsible for transferring methyl groups from SAM to arginine residues in proteins, thereby modulating their function [7]. Aberrant PRMT activity has been associated with progression in various cancers [8]. Consequently, elucidating the role of PRMTs in cell death may provide avenues for devising anti-tumour therapies. The authors found that PRMT5, a type-Ⅱ arginine methyltransferase, directly interacts with RIPK1 and facilitates R606 methylation. The deletion of *Prmt5* resulted in increased cell death upon treatment with TNF, an effect that was abolished in RIPK1 R606K cells. Thus, RIPK1 R606 is specifically methylated by PRMT5 in an SAM-dependent manner.

Liver cirrhosis is a hallmark of chronic hepatitis, alcoholic liver disease, non-alcoholic fatty liver disease, and other conditions. It also serves as a precursor to the development of hepatocellular carcinoma (HCC) [9]. In murine models, *Prmt5* deletion in the liver has been shown to induce liver damage and cirrhosis. Utilizing this model, the authors found that the kinase-inactive RIPK1 could prevent liver damage and associated pathologies, highlighting the physiological significance of R606 methylation in RIPK1 function. This intriguing observation suggests the need for further investigation into the expression and function of PRMTs during the progression from liver damage to cirrhosis and indicates potential therapeutic applications in early stages of cirrhosis.

As mentioned above, RIPK1 activation can also promote necroptosis. Caspase-8 activity inhibits necroptosis, in part by cleaving RIPK1 [10]. Global ablation of caspase-8 causes embryonic lethality in mice, which is prevented by co-ablation of RIPK3 or MLKL. Specific ablation of caspase-8 in liver hepatocytes, however, causes no disease, owing to the absence of RIPK3 expression in the liver. However, under high-fat diet (HFD) conditions, RIPK3 is expressed, and mice lacking liver caspase-8 succumb to loss of liver function. It is therefore possible that in HFD-fed mice, Met deprivation may result in exacerbated liver diseases that cannot be obviated by inhibition of caspase-8 function.

Overall, Chen *et al*. discovered a previously unknown regulation of RIPK1 via Met−SAM metabolism, offering mechanistic insights into cellular behavior under nutrient-stressed conditions. This study also sets the stage for further investigation into metabolic regulation of cell death pathways, and potential therapeutic endeavors.
